# Out-of-pocket expenses and rationing of insulin and diabetes supplies: findings from the 2022 T1International cross-sectional web-based survey

**DOI:** 10.3389/fcdhc.2024.1293882

**Published:** 2024-04-22

**Authors:** Katherine Janine Souris, Elizabeth Pfiester, Axel Thieffry, Yanbing Chen, Katarina Braune, Mridula Kapil Bhargava, Ravjot Samra, Pilar Gómez, Shane O'Donnell

**Affiliations:** ^1^T1International, Greensboro, NC, United States; ^2^T1International, Stroud, United Kingdom; ^3^T1International, Copenhagen, Denmark; ^4^Applied Aviation Sciences Department, Embry-Riddle Aeronautical University, Daytona, FL, United States; ^5^Institute of Medical Informatics, Charité – Universitätsmedizin Berlin, Berlin, Germany; ^6^Diabetes Fighters Trust, Delhi, India; ^7^T1International, Burlington, ON, Canada; ^8^DiabetesLATAM, Panamá, Panama; ^9^School of Sociology, University College Dublin, Dublin, Ireland

**Keywords:** type 1 diabetes, T1D, insulin, rationing, out-of-pocket expenses, COVID-19, health policy, health equity

## Abstract

**Introduction:**

Continue investigating Out-of-Pocket Expenses (OoPEs) and rationing of insulin and diabetes supplies, including impacts of the COVID-19 pandemic, for people with type 1 diabetes (T1D).

**Methods:**

A cross-sectional web-based survey was conducted in English and advertised by T1International’s global network of patient advocates from May through September 2022. Participants provided monthly OoPEs and rationing frequency for insulin and supplies, impacts of the COVID-19 pandemic, and open-ended comments.

**Results:**

In the seven most represented countries, mean monthly OoPEs were highest in the United States, followed by Panama, Canada, and India, and were much lower in the United Kingdom, Germany, and Sweden. OoPEs were highest for participants with partial healthcare coverage, followed by those with no healthcare coverage. The COVID-19 pandemic negatively impacted access and/or affordability of insulin and/or supplies for over half of participants. Globally, 19.5% reported insulin rationing and 36.6% reported rationing glucose testing supplies. Qualitative analysis of open-ended responses identified themes such as ‘mental health impacts’ and ‘limits to life choices.’

**Discussion:**

High OoPEs lead to rationing of insulin and supplies for many people with T1D globally. Healthcare systems improvements and price reductions of insulin and supplies are needed to ensure adequate, equitable access for all.

## 1 Introduction

Without insulin, a person living with type 1 diabetes (T1D) cannot survive (1). Despite its discoverers’ intent that insulin be accessible to all who need it (1–3) and their desire to prevent its exploitation by monopolies (4, 5), it has become a commercial product, grossing huge profits over recent decades (2, 5, 6). More than 100 years after its discovery, many people with T1D die because they cannot access or afford insulin and other essential diabetes supplies (7–9). Others face extreme financial burdens, paying 20-100% or more of monthly income towards diabetes management (10, 11). T1International (https://www.t1international.com/), a non-profit advocating for the rights of people with T1D worldwide, and others have documented the countless deaths resulting from insulin rationing (5, 12, 13), and called for international declarations prioritizing insulin access (14). Market dominance by three insulin manufacturers (5, 7, 15), patent evergreening (16), price discrimination (6), and supply-chain inefficiencies (17, 18) have been cited as contributors to high prices in the absence of governmental regulation (7, 19). In many countries, healthcare systems lack or leave gaps in coverage for diabetes treatment (20–22), contributing to rationing of insulin and supplies (23).

Various studies have investigated diabetes-related out-of-pocket expenses (OoPEs) for particular products or focused on insulin rationing associated with cost in one clinic or region. Health Action International found extreme price variation of glargine products across 47 countries (24). Herkert and colleagues surveyed patients living with T1D from one US clinic and found 26.5% rationed insulin due to costs (25). Analyzing 2021 National Health Interview Survey data, Gaffney and colleagues estimated insulin rationing prevalence among US adults with T1D to be 18.6% (23), and Fang and Selvin found 23.6% of individuals with T1D younger than 65 rationed insulin due to cost (26). T1International aims to add to existing literature through its Out-of-Pocket Expenses (OoPEs) survey, which it has carried out every two years since 2016 (www.t1international.com/access-survey) (10). To our knowledge, this was the first and remains the largest global survey comparing self-reported expenses and rationing of insulin and diabetes supplies.

Using 2020 OoPEs survey data, Pfiester et al. found people with T1D reporting significant OoPEs and rationing of insulin and other diabetes supplies, and negative impacts from the COVID-19 pandemic on access and affordability of supplies (10). Through quantitative and qualitative analysis of 2022 OoPEs survey data, the present study investigates i) the status of OoPEs and rationing among people with T1D, ii) factors contributing to OoPEs and rationing, and iii) the current impact of the COVID-19 pandemic on access and affordability of insulin and diabetes supplies among people with T1D.

## 2 Materials and methods

### 2.1 Survey design and procedures

An anonymous, web-based, cross-sectional survey was conducted in English from May through September 2022 using the Research Electronic Data Capture (REDCap) tool hosted at Cincinnati Children’s Hospital Medical Center (27, 28; *RRID: SCR_003445*) (**Supplementary Data 1**). As previously described (10), this survey was patient-designed. Updated questions included newer insulins and diabetes supplies, and previously omitted categories of expenses. Eight volunteers living with T1D across seven countries pilot tested the survey on various devices, and suggested improvements related to formatting and applicability across country contexts.

Survey introductory text informed individuals that their participation was voluntary and consent was requested before proceeding to survey questions. Participants were asked to select whether they had no coverage for any of their diabetes-related costs, coverage for some of their costs, or coverage for all of their costs, paying nothing out-of-pocket. Participants reported monthly OoPEs in their currency of choice for the following categories: 1) devices, which included insulin pumps and continuous (or flash) glucose monitors (CGM), 2) insulin, 3) pen needles and syringes, and 4) blood glucose testing strips. They also reported the price per product paid for a glucagon emergency shot or nasal spray, if applicable. Additionally, participants could provide free text comments to two open-ended questions (**Supplementary Data 1**). The study was declared low-risk and granted ethical exemption by the Human Research Ethics Committee – Humanities (HREC-HS) of the University College Dublin (HS-E-22-33-ODonnell). T1International and its global volunteers advertised the survey on social media.

### 2.2 Data analysis

Quantitative analyses were conducted within the R statistical framework (https://www.r-project.org;*RRID* *: SCR_001905*). Currencies were converted to USD with the priceR package (https://cran.r-project.org/web/packages/priceR). Distribution of the monthly OoPEs was investigated, and three outlier respondents with more than 5,000 USD per month were removed from further analysis. Per product expenses for glucagon were analyzed separately from monthly OoPEs.

Open-ended responses were analyzed by two of the authors using template analysis (29). First, the researchers familiarized themselves with the data and identified preliminary themes related to the reasons for and impacts of OoPEs and rationing. Through critical dialogue, the researchers created a codebook and then independently coded a subset of 34 data observations to assess intercoder reliability (30). The codebook and code definitions were revised after further dialogue. Subsequently, the researchers deductively analyzed all data using the revised codebook, and iteratively updated it through ongoing dialogues (31). The codebook ultimately consisted of 18 codes with 15 subcodes and identified 6 primary themes.

## 3 Results

### 3.1 Countries represented and healthcare coverage of participants

**Figure 1:**
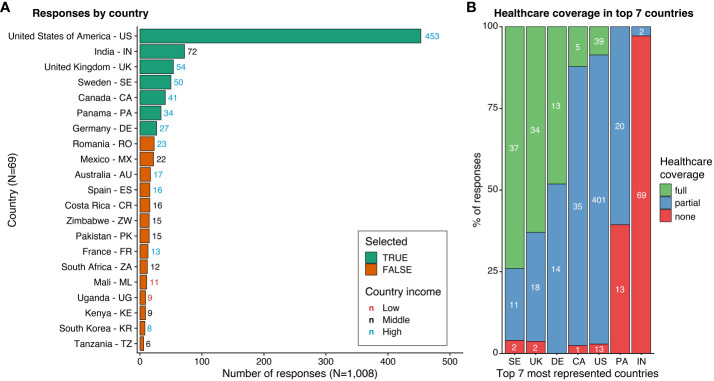
Total responses by country and healthcare coverage in the seven most represented countries. **(A)** Listing of countries (Y-axis) ordered by number of responses (X-axis). Countries are indicated by full name followed by their alpha-2 code. Seven most represented countries selected for further analysis are indicated in green, others in orange. Only countries with more than 4 respondents are shown. **(B)** Ratio of reported healthcare coverage levels (Y-axis, percent) in the seven most represented countries (X-axis). Colors indicate the type of healthcare coverage. White numbers denote the number of responses.

A total of 1,122 responses were recorded. Of these, 28 were removed for lacking explicit consent, 21 for being under 18 years old, 54 for omitting currency of choice, 5 for missing country information, 3 due to outlying monthly costs (see section 2.2), and 3 were manually identified as duplicates, leaving a total of 1,008 records representing 69 countries (**Figure 1A**).

Of the 1,008 responses included in analyses, 724 (71.8%) identified as female, 766 (76%) were adults living with T1D, and 223 (22.1%) were parents/caregivers (**Table 1**). A threshold of 25 responses was used to determine countries in the comparison sample, which resulted in a total of 731 responses from 7 countries: the United States (US), India, the United Kingdom (UK), Sweden, Canada, Panama, and Germany. Healthcare coverage composition of participants could be described as mostly full coverage in Sweden (74%) and the UK (63%), full (48.1%) and partial (51.9%) coverage in Germany, mostly partial coverage in the US (88.5%) and Canada (85.4%), mostly partial coverage (60.6%) with the remaining having no coverage (39.4%) in Panama, and mostly no coverage (97.2%) in India (**Figure 1B**).

**Table 1:** Demographic characteristics of all participants worldwide and in the seven most represented countries.

Characteristic	Answer	Worldwide (N=1,008)	Seven Most Represented Countries (N=731)
Gender	Female	724 (71.8%)	533 (72.9%)
	Male	262 (26%)	178 (24.4%)
	Other	12 (1.2%)	12 (1.6%)
	Prefer not to answer	10 (1.0%)	8 (1.1%)
Connection to T1D	Patient	766 (76%)	562 (76.9%)
	Parent/Caregiver	223 (22.1%)	157 (21.5%)
	Partner	13 (1.3%)	10 (1.4%)
	Healthcare Provider	4 (0.4%)	0 (0.0%)
	Prefer not to answer	2 (0.2%)	2 (0.3%)
Country income level	High	768 (76.2%)	659 (90.2%)
	Middle	211 (20.9%)	72 (9.8%)
	Low	29 (2.9%)	0 (0.0%)

### 3.2 OoPEs in the seven most represented countries

**Figure 2:**
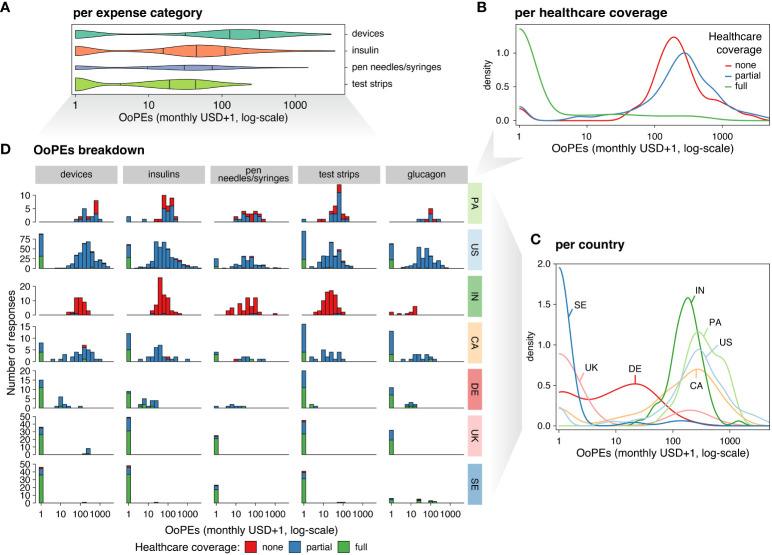
Overview of Out-of-Pocket Expenses (OoPEs) in the seven most represented countries. **(A)** Violin plot of self-reported OoPEs (X-axis) indicated in USD (pseudocount: +1, log-scale), for devices, insulin, pen needles and/or syringes, and testing strips (Y-axis). The strips category (green) includes blood glucose testing strips. The devices category (teal) comprises insulin pumps and CGMs. The insulin category (orange) encompasses short-acting, long-acting, mixed-types, and other types of insulins. Violin ticks indicate quantiles (25%, 50%, and 75% from left to right, respectively) and areas are proportional to the number of responses. **(B)** Density distribution of OoPEs (X-axis, as in A) per healthcare coverage level (colors). **(C)** Density distribution of Out-of-Pocket Expenses (X-axis, organized as in A) per country (colors). **(D)** Breakdown of OoPEs (organized as in A) per expense category (columns), country (rows), and healthcare coverage level (bar colors). Y-axis indicates the number of respondents.

Participants in the seven most represented countries with partial healthcare coverage reported the highest mean monthly OoPEs (434.9 USD; **Figure 2B**), roughly 90 USD higher than those with no healthcare coverage (**Figure 2B**; **Supplementary Data 2, Supplementary Table 1**). Participants with full healthcare coverage reported the lowest mean monthly OoPEs (21.2 USD), over 300 USD less than those with no healthcare coverage and over 400 USD less than those with partial healthcare coverage.

The highest mean monthly OoPEs were reported for devices (**Figures 2A, D**), and standard deviation (SD) was highest in this category (**Supplementary Data 2, Supplementary Table 2**). The second highest mean monthly OoPEs were reported for insulin, with more individuals reporting expenses in this category than any other, independent of healthcare coverage and country (**Figures 2A, D**). Monthly OoPEs for pen needles and syringes were higher than those for test strips but were reported by a smaller percentage of individuals. Mean per-product expenses for glucagon were 57.6 USD (**Figure 2D**; **Supplementary Data 2, Supplementary Table 2**).

Participants from the US reported the highest mean monthly OoPEs (471.1 USD), followed by Panama, Canada, and India (**Figure 2C**). Very low mean monthly OoPEs were reported in the UK, Germany, and Sweden (**Figure 2C**). Of note, SD of OoPEs was highest in the US, 396.4 USD greater than any other country, and median expenses in Panama were greater than in the US (**Supplementary Data 2, Supplementary Table 3**). Median OoPEs were also greater in Germany than in the UK. OoPEs towards test strips were highest in Panama as compared to other countries, independent of healthcare coverage level (**Figure 2D**).

Device use was common across the seven countries, with 87.6% of participants reporting CGM use and 63.2% reporting insulin pump use (**Figure 2D**). CGM use was reported by over 90% of participants in Germany, Sweden, Canada, the US, and the UK, and by over 50% of participants in Panama and India (**Figure 2D**). Insulin pump use was reported by over 70% of participants in the US, over 60% of participants in Germany, Sweden, and Canada, and over 50% of participants in the UK. Nearly 30% of participants in Panama and nearly 17% of participants in India reported insulin pump use (**Figure 2D**).

### 3.3 Rationing of insulin and glucose testing supplies

#### 3.3.1 Worldwide rationing

**Figure 3:**
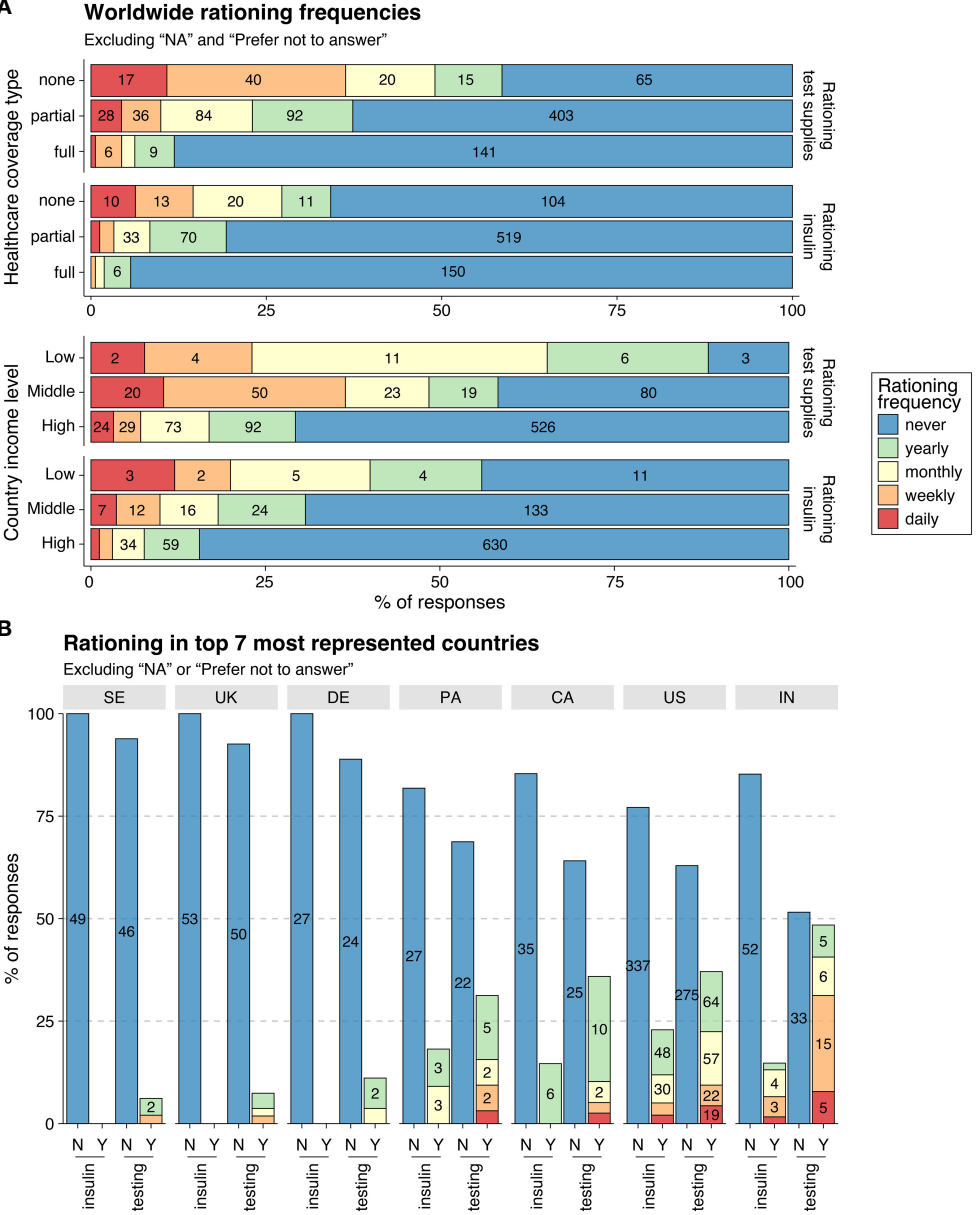
Rationing of insulin and glucose testing supplies. **(A)** Worldwide overview of rationing frequencies (bar colors) according to healthcare coverage level (top) and country income level (bottom), further divided by rationing of testing glucose levels (top bars), and rationing/skipping insulin due to cost (bottom bars). X-axis indicates the percentage of responses and numbers are shown within each bar. **(B)** Rationing in the seven most represented countries (columns), with bar colors as in **(A)** X-axis shows whether rationing concerned insulin intake or glucose testing (N, No; Y, Yes).

Among all 1,008 participants worldwide, 19.5% rationed insulin and 36.6% rationed glucose testing supplies, while among those living in low-income countries, 56% rationed insulin and 88.5% rationed glucose testing supplies at some point over the past year (**Figure 3A**). There was a visible negative correlation between the level of healthcare coverage and rationing frequency, both for testing supplies and insulin (**Figure 3A**). Rationing was most prevalent for individuals with no healthcare coverage, with 34.2% rationing insulin and 58.6% rationing testing supplies at some point over the past year (**Figure 3A**).

#### 3.3.2 Rationing in the seven most represented countries

Insulin rationing was reported by 21.2% of participants across the US, Panama, India, and Canada, and by no participants in Sweden, the UK, or Germany (**Figure 3B**). The US had the highest percentage of participants rationing insulin at some point over the previous year, followed by Panama, India, and Canada (**Figure 3B**). The frequency of insulin rationing varied across the four countries. No participants from Canada rationed more often than yearly and no participants from Panama rationed more often than monthly, while participants in the US and India rationed weekly or more (**Figure 3B****;** **Supplementary Data 2, Supplementary Table 4A**).

Overall, rationing of glucose testing supplies was more common than insulin rationing, with 32.3% of participants across the seven countries reporting rationing testing supplies at some point over the past year (**Figure 3B****).** However, the prevalence and frequency of rationing varied widely across countries. India had the highest percentage of participants rationing testing supplies at some point, followed by the US, Canada, and Panama (**Figure 3B**). A much smaller percentage of participants rationed testing supplies at some point in Germany, the UK, and Sweden (**Figure 3B**). No participants in Germany rationed more often than monthly, whereas in the UK and Sweden, a small percentage of participants rationed weekly (**Figure 3B**; **Supplementary Data 2, Supplementary Table 4B**). While rationing glucose testing supplies at some point in the US and Canada was similar, a higher percentage of participants in the US rationed monthly or more often compared to Canada (**Figure 3B**; **Supplementary Data 2, Supplementary Table 4B**). India had the highest percentage of participants rationing weekly or more.

### 3.4 Impact of COVID-19 in the seven most represented countries

Self-reported negative impacts of the COVID-19 pandemic on access and/or affordability of insulin and/or supplies varied across countries. The highest proportion of affected participants were from Panama, with 67.7% reporting challenges (**Supplementary Data 2, Supplementary Figure 1**). Similarly, participants from the US (58.5%) and India (56.9%) were significantly impacted. In Canada, roughly half of participants reported access and/or affordability challenges from the pandemic, while much smaller percentages of participants from Germany (29.6%), the UK (25.9%), and Sweden (14%) experienced negative impacts (**Supplementary Data 2, Supplementary Figure 1**).

### 3.5 Qualitative analysis results

Qualitative analysis of responses to open-ended questions identified six primary themes: “Healthcare system improvements needed”; “Problems with insurance and national or state healthcare coverage”; “Strengths of national or state healthcare coverage”; “Other diabetes-related expenses”; “Mental health impacts”; “Limits to life choices”; and “Human Rights”. We detail each theme below using participant quotes.

#### 3.5.1 Healthcare system improvements needed

Overwhelmingly, participants called on their governments for affordable access to insulin and diabetes supplies, and spoke to the resulting cost-saving potential:

*“Make CGM access available for everyone! It will save you money in healthcare hospital visits for people with T1D.”* (person with T1D, partial healthcare coverage, Canada)

In India, multiple participants wanted T1D classified as a disability to ensure healthcare coverage for diabetes supplies: *“T1D is not covered by health insurance in India. Consider T1D as a disability and support the person.”* (parent/caregiver of a child with T1D, no healthcare coverage, India)

Inaccessibility compounded unaffordability for many participants in Panama: *“In my region[, it] is too expensi[ve] or do not [exist] the state of the art products for diabetes. So we [cannot] give our children the best treatment[…]”* (parent/caregiver of a child with T1D, partial healthcare coverage, Panama)

#### 3.5.2 Limits to life choices

Participants sacrificed other basic needs due to high diabetes-related OoPEs: *“Stop making me choose between buying diabetes supplies and food.”* (person with T1D, partial healthcare coverage, US)

OoPEs towards diabetes dictated participants’ choices apart from diabetes management:

*“I also chose which province to live in based on which has the best coverage for diabetes supplies.”* (person with T1D, full healthcare coverage, Canada)

One participant in the US with partial healthcare coverage described how high OoPEs affected their family decisions: *“I had to put off trying for another child because my insulin was so expensive[. W]e were not able to afford another child along with the cost of my diabetes.”*

#### 3.5.3 Mental health impacts

Participants described the fear, stress, and anxiety of being unable to afford and/or access supplies:

*“There is also the emotional cost of the stress of worrying if supplies will last or arrive in time.” (person with T1D, partial healthcare coverage, US)*

*“Managing diabetes is both mentally and financially draining…”* (parent/caregiver of a person with T1D, no healthcare coverage, India)

Participants discussed the varying degree to which the need for mental health care is recognized:

*“Sweden may be one of the few countries in the world that covers nearly all diabetes-related costs. What many of us miss though [is] more attention to interrelated conditions that [make] it difficult to get your diabetes under control, such as mental health…”* (person with T1D, full healthcare coverage, Sweden)

#### 3.5.4 Problems with insurance and national or state healthcare coverage

Many participants from the US described insurance as extremely expensive and burdensome: *“40% of my monthly paycheck goes towards insurance and I still have copays and [out-of-pocket] costs.”* (person with T1D, partial healthcare coverage, US)

*“Why do I have to plead, negotiate, yell, follow up, and chase down the drugs and supplies [I] need to stay alive every month?”* (person with T1D, partial healthcare coverage, US)

Others in the US were unable to access insurance:

*“…We make too much to qualify for any assistance, yet not enough for Private insurance. I’m tired of feeling sick because I [cannot] afford the insulin I should be on or see a doctor.”* (person with T1D, no healthcare coverage, US)

A parent/caregiver of a child with T1D with partial coverage from Panama feared their child losing healthcare coverage:

*“The coverage of [diabetes] supplies is covered by [the] government just because my child is underage (18 years)[. O]nce he is over 18 years[,] he will [no] longer have [these] supplies as [a] priority.”*

One person living with T1D from Canada with partial healthcare coverage spoke to its limits: “*I am fortunate to have 80% coverage, however[,] I have to pay for it upfront and I am [reimbursed] in a week. That week makes a big difference[,] especially when supplies are only dispensed for 30 days. I know I am one of the lucky ones[,] but I am struggling.”*

#### 3.5.5 Strengths of national or state healthcare coverage

Some participants from countries with universal healthcare described their healthcare coverage positively and as comprehensive:

*“I am always really appreciative that where I live[,] there will always be a safety net for my insulin needs [through] the government. I know I’m incredibly lucky to be a diabetic in Canada and not another country without public healthcare.”* (person with T1D, full healthcare coverage, Canada)

Others acknowledged that their healthcare comes at a cost through taxes:

*“The health care system here is socially funded but the tax (National Insurance) premium that pays for this is 23% of my gross income. The supplies are not free, they are free at the point of delivery[…]”* (person with T1D, full healthcare coverage, UK)

In the US, a few participants with full healthcare coverage spoke positively of specific national or state programs:

*“My daughter receives California Children’s Services[,] which covers her for anything diabetes related … Dexcom is covered as a pharmacy benefit by Medi-cal[,] making all these things easily accessible.”* (parent/caregiver of a child with T1D, full healthcare coverage, US)

#### 3.5.6 Other diabetes-related expenses

Participants shouldered diabetes expenses not captured in their reported OoPEs, not limited to hypoglycemia treatment, alcohol swabs, the cost of mental healthcare, and specialist visits and fees:

*“We have to do quarterly lab tests for my kid to check the progression and onset of other autoimmune problems. It costs us around 100,000 INR [1,210.47 USD] per year.”* (parent/caregiver of a child with T1D, no healthcare coverage, India)

Participants spoke to how diabetes-related complications resulted from cost-related rationing:

*“I had to ration my insulin and diabetes supplies when I was uninsured and underinsured. There were insurance plans I had that did not cover insulin pump supplies. My health deteriorated and now I’m in end stage renal failure on dialysis.”* (person with T1D, partial healthcare coverage, US)

#### 3.5.7 Human rights

Many participants used human rights-based language to express their beliefs:

*“Insulin is a basic human right. I know I’m privileged, and I have never known the pain of rationing, but I will keep fighting for our right to live as long as it takes.”* (person with T1D, partial healthcare coverage, US)

*“It is not a choice. It is literally life or death.”* (person with T1D, partial healthcare coverage, Canada)

When speaking about the need for more affordable supplies, many participants expressed a desire for equity: *“…Individuals with diabetes should not be discriminated against by policies that support insulin pumps for specific age groups and not others. And no one with diabetes should ever have to choose between taking their life saving medication, feeding their families or paying rent.”* (person with T1D, partial healthcare coverage, Canada)

## 4 Discussion

This study highlights the significant financial burden that people with T1D face in the absence of full healthcare coverage and associated rationing of insulin and other diabetes supplies. Even in this sample of participants from mostly high-income countries (32), a significant number rationed insulin and glucose testing supplies.

Study results demonstrated global inequities in OoPEs and subsequent rationing. In the seven most represented countries, OoPEs and rationing were much higher for those with both partial and no healthcare coverage as compared to those with full healthcare coverage. Participants in Germany, the UK, and Sweden had low to no OoPEs, reported no insulin rationing, and a lower percentage of participants rationed testing supplies, while participants in the US, Panama, Canada, and India shouldered high monthly OoPEs (>200 USD) and reported associated rationing of insulin and testing supplies. While the cross-sectional nature of this survey limits direct comparison of 2022 results with those from 2020, US participants continued to report rates of insulin rationing over 20% and similarly high rates of glucose testing supply rationing nearing 40% (10). In India, a lower-middle income country (32), people with T1D are not protected under the Rights of Persons with Disabilities Act of 2016 (33), leaving them without assistance for costs (34). Canada, unlike other countries with universal healthcare, lacks universal pharmacare, which can lead to higher medication costs for individuals and rationing of supplies (35). In Panama, access to newer insulins is limited and supply shortages are common (36). While the US is one of the ten richest countries in the world, US participants reported the highest mean monthly OoPEs. The US also exhibited the highest SD of OoPEs, corroborating research that demonstrates vast inequities in healthcare access in the US (23, 26).

In the seven most represented countries, those with no healthcare coverage for diabetes-related expenses reported slightly lower OoPEs than those with partial healthcare coverage. This may be explained by the higher incidence of rationing among participants with no healthcare coverage, since people often ration to spend less. Because healthcare systems and subsequent coverage for health expenses vary markedly from country to country, it is likely that the partial coverage category encompasses a wide range of coverage levels. Partial healthcare coverage may also put more advanced technology in reach, even while healthcare-related expenses absorb funds for other necessities and limit choices, which was evidenced by our qualitative findings. Quantitative results demonstrated lower device usage in India and Panama, where higher percentages of participants lacked healthcare coverage, and qualitative results support unavailability of technology, particularly expressed by participants in Panama, and unaffordability when available.

While the COVID-19 pandemic disrupted access to insulin and supplies for over half of participants more than two years after its onset, increased availability of therapeutics and vaccines may have decreased the burden on healthcare systems, and therefore contributed to more stable supply chains as compared to earlier years. Furthermore, unlike in 2020 when travel bans and restrictions were more prevalent, people could travel between countries and states in 2022 more freely, meaning that increased access via medical tourism could have mitigated potential shortages or rationing that was observed during earlier stages of the pandemic (37).

As in 2020 (10), in our 2022 sample rationing testing supplies was more common than insulin rationing. Recommended frequency of self-monitoring is lacking in many countries. For example, in India, T1D management guidelines from the Indian Council for Medical Research instruct providers to adapt their recommendations to a patient’s financial constraints, rather than promoting an essential minimum daily number of glucose tests (38). While many local diabetes organizations are advocating for patient rights in their communities, global entities like the World Health Organization should advocate for an international standard to ensure adequate provision of glucose monitoring supplies for people with T1D.

Our qualitative analysis deepened understanding of the variability in healthcare coverage that many participants experienced, particularly in countries without comprehensive universal healthcare. The ability to access life-sustaining medication for people with T1D depends on their current healthcare coverage, which can be highly variable in terms of what is covered and to what extent (39). Many responses related to conditional coverage, meaning participants may have rationed in the past or be at risk of rationing in the future. The connection between rationing and out-of-target glycemic outcomes has been documented (25), which in turn increases an individual’s risk of diabetes-related complications (40).

Challenges in healthcare systems apart from high OoPEs may contribute to rationing. For example, in Canada, variability in how prescriptions are written and interpreted between provinces and territories can lead to delays in receiving supplies (35). In India, stigma related to social status and gender (41) and healthcare provider attitudes (42) may contribute to rationing for some individuals.

Strengths of this study include patient involvement at every stage, from design through analysis, and increased country representation and larger sample sizes respectively from countries other than the US, as compared to 2020 (10). Limitations include a sample of participants that was likely relatively socioeconomically advantaged, as evidenced by a higher than expected percentage of participants across the seven most represented countries reporting the use of more advanced diabetes technologies. Over half of participants in India reported CGM use, despite limited availability and barriers to use in the region (43, 44). In a recent study using data from the T1D Exchange Registry, 48% of people with T1D (N=11,469) from eight US care centers were CGM users (45), compared to 91.8% of US participants in our sample. In short, rationing of both insulin and testing supplies by people with T1D globally is likely substantially higher than our results indicate, especially for less socioeconomically advantaged individuals.

As in years past, the survey was only available electronically and in English, which may have introduced selection bias and led to a sample favoring those of higher incomes and higher access to care. Qualitative insights should be understood in the context of our specific sample, including the effects of potential bias, and are not generalizable. Furthermore, inherent bias may exist in qualitative data, as participants experiencing more challenges may be more likely to respond to open-ended questions. Translating the survey into other languages and promoting electronic and paper delivery by local community members can increase representation of non-English speakers and individuals with lower incomes and lower access to care in future versions. Other limitations include that the self-report nature of the study, while vital to its design, introduces the potential for recall bias and inaccuracies in reported OoPEs and rationing frequency. Additionally, the cross-sectional design of the study prohibits inference of causality between OoPEs and rationing frequency.

Addressing inequities in healthcare coverage and access to essential treatments should be of top priority for healthcare systems. T1International and its global community of activists have advocated for the past decade for cost-reductions of insulin and other diabetes supplies at the manufacturer level, calling for the rights of patients over unrestricted profits. T1International has created freely accessible tools for advocates wishing to become involved in the fight for #insulin4all, such as its Advocacy Toolkit (https://www.t1international.com/toolkit/), and trainings available on its YouTube channel (https://www.youtube.com/@t1international). Further research is needed to explore the full extent of rationing, including of food and other necessities, and the restrictions that high OoPEs impose on people living with T1D throughout many areas of their lives. The resulting improved understanding of the financial burden faced by individuals with T1D should inform policy changes, including governmental regulation of industry, and targeted multi-stakeholder interventions, to improve access to affordable diabetes care worldwide.

## 5 Conclusion

The financial cost of maintaining basic survival for people with T1D is excessively high and is leading to rationing of insulin and diabetes supplies for many. Rationing of insulin remains a significant issue worldwide, affecting 1 in 5 people in our sample, and rationing of glucose testing supplies is also of concern. This study supports the need for continued advocacy by T1International and others unbiased by pharmaceutical and industry funding, healthcare systems improvements at the governmental level, and price reductions of insulin and diabetes supplies at the global level to ensure adequate, equitable access to insulin and diabetes supplies, including advanced technologies, for all.

## Data availability statement

The original contributions presented in the study are publicly available. This data can be found on the GitHub repository here: https://github.com/athieffry/T1International-OoPE-survey-2022.

## Ethics statement

The requirement of ethical approval for the studies involving humans was waived by the Human Research Ethics Committee – Humanities (HREC-HS) of the University College Dublin (HS-E-22-33-ODonnell). The study was conducted in accordance with the local legislation and institutional requirements. The participants provided their written informed consent to participate in this study.

## Author contributions

KS: Formal analysis, Investigation, Project administration, Resources, Writing – original draft, Writing – review & editing, Methodology, Supervision. EP: Conceptualization, Investigation, Writing – original draft, Writing – review & editing, Resources, Project administration, Supervision. AT: Data curation, Formal analysis, Methodology, Visualization, Writing – original draft, Writing – review & editing. YC: Formal analysis, Methodology, Resources, Writing – original draft, Writing – review & editing. KB: Resources, Writing – original draft, Writing – review & editing. MKB: Writing – original draft, Writing – review & editing, Resources. RS: Resources, Writing – original draft, Writing – review & editing. PG: Resources, Writing – original draft, Writing – review & editing. SO: Supervision, Writing – review & editing, Project administration, Writing – original draft.
