# Supplementary material for: Out-of-pocket expenses and rationing of insulin and diabetes supplies: findings from the 2022 T1International cross-sectional web-based survey
Source: Front Clin Diabetes Healthc. 2024 Apr 22;5:1293882. doi: 10.3389/fcdhc.2024.1293882 (PMC11070566; doi:10.3389/fcdhc.2024.1293882)
Supplement: Supplementary file 1 [file DataSheet_1.pdf]

Type 1 Diabetes Out-of-Pocket Cost Survey

You are invited to take part in an online survey about your type 1 diabetes (T1D) costs. It should take approximately 20-25 minutes to complete. You must be at least 18 years old to complete the survey. Primary caregivers (usually parents) may complete the survey for individuals under the age of 18.

T1International is conducting this survey. Your participation is completely voluntary. You will receive no money or other financial reward by participating in this survey.

Your responses will help build a global picture of diabetes costs, and support advocacy for better access and policy change. There are minimal risks to your privacy in completing this survey. We will not request any information that leads to your identity. Should you provide any information that could lead to your identify in free text fields (open-ended questions), this will be removed before analysis.

If you have questions, please email T1International at contact@t1international.com.

By clicking "agree" below, you are e-signing this form and agree to the following:

I have read this information and have had time to consider whether to take part in this study. I understand that my participation is voluntary (it is my choice) and that I am free to withdraw from the research study at any time without disadvantage. I agree that the data can be used in the publication of scientific and research work. I agree that the information collected in this study will be stored in a protected archive where it may be available for future research. I understand that by authorizing the use of my personal data obtained in this survey, data privacy laws might not apply or no longer protect my information. I have read this consent form. I understand that I can refuse to participate in this project. I have taken time to think carefully about my decision to participate. I freely consent to share my data with this research project.

◯ I have read the above information and I agree to participate
◯ I am not interested

I am 18 years old or older	◯ Yes<br>◯ No

In this survey, we will be asking about your out-of-pocket costs. We will ask you to select the currency you use to calculate costs.

Out-of-pocket costs are the amount you pay from your own funds, including any taxes, fees, or other charges. For more definitions, visit our Glossary.

Please complete a separate survey for each person in your household with type 1 diabetes. If completing on behalf of someone with T1D, note that references to "you/your" refer to the person with T1D.

What is your connection to type 1 diabetes?
(If completing on behalf of someone with T1D, note that references to "you/your" refer to the person with T1D)

◯ I have type 1 diabetes
◯ My child has type 1 diabetes
◯ My spouse/partner/significant other has type 1 diabetes
◯ I am a medical professional completing survey on behalf of a specific patient with type 1 diabetes
◯ Prefer not to answer

What is your gender?

◯ Male
◯ Female
◯ Non-binary
◯ Other _______
◯ Prefer not to answer

Do you identify as transgender?

◯ Yes
◯ No
◯ Prefer not to answer

What country do you live in?

◯ Afghanistan
◯ Albania
◯ Algeria
◯ Andorra
◯ Angola
◯ Antigua and Barbuda
◯ Argentina
◯ Armenia
◯ Aruba
◯ Australia
◯ Austria
◯ Azerbaijan
◯ Bahamas, The
◯ Bahrain
◯ Bangladesh
◯ Barbados
◯ Belarus
◯ Belgium
◯ Belize
◯ Benin
◯ Bhutan
◯ Bolivia
◯ Bosnia and Herzegovina
◯ Botswana
◯ Brazil
◯ Brunei
◯ Bulgaria
◯ Burkina Faso
◯ Burma
◯ Burundi
◯ Cabo Verde
◯ Cambodia
◯ Cameroon
◯ Canada
◯ Cayman Islands
◯ Central African Republic
◯ Chad
◯ Chile
◯ China
◯ Colombia
◯ Comoros
◯ Congo, Democratic Republic of the
◯ Congo, Republic of the
◯ Costa Rica
◯ Cote d'Ivoire
◯ Croatia
◯ Cuba
◯ Curacao
◯ Cyprus
◯ Czechia
◯ Denmark
◯ Djibouti
◯ Dominica
◯ Dominican Republic
◯ East Timor
◯ Ecuador
◯ Egypt
◯ El Salvador
◯ Equatorial Guinea
◯ Eritrea
◯ Estonia
◯ Ethiopia
◯ Fiji
◯ Finland
◯ France
◯ Gabon
◯ Gambia, The

◯ Georgia
◯ Germany
◯ Ghana
◯ Greece
◯ Grenada
◯ Guatemala
◯ Guinea
◯ Guinea-Bissau
◯ Guyana
◯ Haiti
◯ Holy See
◯ Honduras
◯ Hong Kong
◯ Hungary
◯ Iceland
◯ India
◯ Indonesia
◯ Iran
◯ Iraq
◯ Ireland
◯ Israel
◯ Italy
◯ Jamaica
◯ Japan
◯ Jordan
◯ Kazakhstan
◯ Kenya
◯ Kiribati
◯ Korea, North
◯ Korea, South
◯ Kosovo
◯ Kuwait
◯ Kyrgyzstan
◯ Laos
◯ Latvia
◯ Lebanon
◯ Lesotho
◯ Liberia
◯ Libya
◯ Liechtenstein
◯ Lithuania
◯ Luxembourg
◯ Macau
◯ Macedonia
◯ Madagascar
◯ Malawi
◯ Malaysia
◯ Maldives
◯ Mali
◯ Malta
◯ Marshall Islands
◯ Mauritania
◯ Mauritius
◯ Mexico
◯ Micronesia
◯ Moldova
◯ Monaco
◯ Mongolia
◯ Montenegro
◯ Morocco
◯ Mozambique
◯ Namibia
◯ Nauru
◯ Nepal
◯ Netherlands
◯ New Zealand
◯ Nicaragua
◯ Niger
◯ Nigeria
◯ North Korea
◯ Norway

◯ Oman
◯ Pakistan
◯ Palau
◯ Palestinian Territories
◯ Panama
◯ Papua New Guinea
◯ Paraguay
◯ Peru
◯ Philippines
◯ Poland
◯ Portugal
◯ Qatar
◯ Romania
◯ Russia
◯ Rwanda
◯ Saint Kitts and Nevis
◯ Saint Lucia
◯ Saint Vincent and the Grenadines
◯ Samoa
◯ San Marino
◯ Sao Tome and Principe
◯ Saudi Arabia
◯ Senegal
◯ Serbia
◯ Seychelles
◯ Sierra Leone
◯ Singapore
◯ Sint Maarten
◯ Slovakia
◯ Slovenia
◯ Solomon Islands
◯ Somalia
◯ South Africa
◯ South Korea
◯ South Sudan
◯ Spain
◯ Sri Lanka
◯ Sudan
◯ Suriname
◯ Swaziland
◯ Sweden
◯ Switzerland
◯ Syria
◯ Taiwan
◯ Tajikistan
◯ Tanzania
◯ Thailand
◯ Timor-Leste
◯ Togo
◯ Tonga
◯ Trinidad and Tobago
◯ Tunisia
◯ Turkey
◯ Turkmenistan
◯ Tuvalu
◯ Uganda
◯ Ukraine
◯ United Arab Emirates
◯ United Kingdom
◯ United States of America
◯ Uruguay
◯ Uzbekistan
◯ Vanuatu
◯ Venezuela
◯ Vietnam
◯ Yemen
◯ Zambia
◯ Zimbabwe

Which of these do you identify with? (Choose all that apply)
Note: our surveys are global, so we have chosen very broad categories to cover as many identities as possible, with the option to write in yours specifically.

☐ Asian
☐ Black/African/African American
☐ Hispanic/Latino/Mestizo
☐ Indigenous
☐ Middle Eastern/North African
☐ White/Caucasian
☐ Other ______
☐ Prefer not to answer

What currency do you use?

◯ Australian Dollar (AUD)
◯ Bosnia-Herzegovina Convertible Marka (BAM)
◯ Canadian Dollar (CAD)
◯ Chinese Renminbi (CNH)
◯ Costa Rican Colón (CRC)
◯ Euro (EUR)
◯ Ghanaian Cedi (GHC)
◯ Hong Kong Dollar (HKD)
◯ Indian Rupee (INR)
◯ Israeli New Shekel (ILS)
◯ Japanese Yen (JPY)
◯ Lebanese Pound (LBP)
◯ Mexican Peso (MXN)
◯ New Zealand Dollar (NZD)
◯ Pakistani Rupee (PKR)
◯ Panamanian Balboa (PAB)
◯ Pound Sterling (GBP)
◯ Swedish Krona (SEK)
◯ Swiss Franc (CHF)
◯ Tanzanian Shilling (TSH)
◯ US Dollar (USD)
◯ Zimbabwe Dollar (ZWD)
◯ Your Currency

If your currency, please specify

____
(If selecting "Your Currency", please write your currency in the text box or we cannot calcuate your costs.)

Do you have health coverage that covers the cost of your diabetes medication and supplies?
(Choose the answer the best describes your situation)

◯ No, there is no coverage for any of my costs
◯ Yes, there is health coverage for some of my costs
◯ Yes, there is health coverage for all of my costs (so I do not pay anything out of pocket)
◯ Prefer not to answer

Do you receive any help to pay for your insulin, diabetes supplies, or care?
(Choose all that apply)

☐ No
☐ Yes, support from family and friends
☐ Yes, charities/religious/non-profit programs
☐ Yes, donations (including online platforms like GoFundMe)
☐ Yes, government assistance/benefit programs
☐ Yes, pharmaceutical company assistance programs
☐ Other ______
☐ Prefer not to answer

In the past year, have you had to do any of the following to pay for your out-of-pocket costs for your medication and/or supplies? (Choose all that apply)

☐ None of these
☐ Use savings
☐ Borrowed money
☐ Sold assets
☐ Other ______
☐ Prefer not to answer

Has the COVID-19 pandemic affected your access to insulin and diabetes supplies?

☐ No change
☐ Access to supplies have been delayed or disrupted
☐ Access to insulin has been delayed or disrupted
☐ Prices have gone up
☐ Prices have gone down
☐ Unsure
☐ Prefer not to answer

In what form do you get your insulin?
(Choose all that apply)

☐ Insulin vials
☐ Insulin pens
☐ Inhaled insulin
☐ Other ______
☐ Prefer not to answer

How do you administer the insulin?
(Choose all that apply)

☐ Syringes
☐ Pens with pen needles
☐ Insulin pump
☐ Other ______
☐ Prefer not to answer

How much do you pay in [currency] for a 1 month supply of pen needles and/or syringes?

____

Please select all insulins you currently use.
(Choose all that apply)

☐ Humalog U-100
☐ Humalog U-200
☐ Novolog/Novorapid
☐ Apidra
☐ Admelog
☐ Lyumjev
☐ Humulin
☐ Novolin (Regular)
☐ Fiasp
☐ Velosulin
☐ Lantus/ Toujeo
☐ Levemir
☐ Tresiba U-100
☐ Tresiba U-200
☐ Basaglar
☐ NPH
☐ Humulin 70/30
☐ Humulin 50/50
☐ Humalog 75/25
☐ Novolin 70/30
☐ Novolog 70/30
☐ Mixtard
☐ Rezvoglar
☐ Afrezza
☐ Semglee
☐ Other ______
☐ Prefer not to answer

How many vials of short-acting insulin (e.g. Humalog, Novolog/Novorapid, Apidra, Humulin, Novolin/Regular, Velosulin, Fiasp, Admelog, or Lyumjev) do you typically use per month?

○ 1
○ 2
○ 3
○ 4
○ 5
○ 6
○ 7
○ 8+
○ Prefer not to answer

How many pens of short-acting insulin (e.g. Humalog, Novolog/Novorapid, Apidra, Humulin, Novolin/Regular, Velosulin, Fiasp, Admelog, or Lyumjev) do you typically use per month?

○ 1
○ 2
○ 3
○ 4
○ 5
○ 6
○ 7
○ 8+
○ Prefer not to answer

How much do you pay out of pocket in [currency] for a 1 month supply of short-acting insulin (e.g. Humalog, Novolog/Novorapid, Apidra, Humulin, Novolin/Regular, Velosulin, Fiasp, Admelog, or Lyumjev)?

____
(Number only)

How many vials of intermediate or long-acting insulin (e.g. Lantus/Toujeo, Levemir, Tresiba, Basaglar, NPH, Semglee, or Rezvoglar) do you typically use per month?

◯ 1
◯ 2
◯ 3
◯ 4
◯ 5
◯ 6
◯ 7
◯ 8+
◯ Prefer not to answer

How many pens of intermediate or long-acting insulin (e.g. Lantus/Toujeo, Levemir, Tresiba, Basaglar, NPH, Semglee, or Rezvoglar) do you typically use per month?

◯ 1
◯ 2
◯ 3
◯ 4
◯ 5
◯ 6
◯ 7
◯ 8+
◯ Prefer not to answer

How much do you pay out of pocket in [currency] for a 1 month supply of intermediate or long-acting insulin (e.g. Lantus/Toujeo, Levemir, Tresiba, Basaglar, NPH, Semglee, or Rezvoglar)?

____
(Number only)

How many vials of mixed insulin (e.g. Humulin 70/30, Humulin 50/50, Humalog 75/25, Novolin 70/30, Mixtard or Novolog 70/30) do you typically use per month?

◯ 1
◯ 2
◯ 3
◯ 4
◯ 5
◯ 6
◯ 7
◯ 8+
◯ Prefer not to answer

How many pens of mixed insulin (e.g. Humulin 70/30, Humulin 50/50, Humalog 75/25, Novolin 70/30, Mixtard or Novolog 70/30) do you typically use per month?

◯ 1
◯ 2
◯ 3
◯ 4
◯ 5
◯ 6
◯ 7
◯ 8+
◯ Prefer not to answer

How much do you pay out of pocket in [currency] for a 1 month supply of mixed insulin (e.g. Humulin 70/30, Humulin 50/50, Humalog 75/25, Novolin 70/30, Mixtard or Novolog 70/30)?

____
(Number only)

How many vials of other insulin type do you typically use per month?

◯ 1
◯ 2
◯ 3
◯ 4
◯ 5
◯ 6
◯ 7
◯ 8+
◯ Prefer not to answer

How many pens of other insulin type do you typically use per month?

◯ 1
◯ 2
◯ 3
◯ 4
◯ 5
◯ 6
◯ 7
◯ 8+
◯ Prefer not to answer

How much do you pay out of pocket in [currency] for a 1 month supply of other insulin type?

____
(Number only)

How often do you have to ration or NOT give yourself insulin due to cost?

◯ Never
◯ At least once per year
◯ At least once per month
◯ At least once per week
◯ Every day
◯ Prefer not to answer

What type of insulin pump do you use?

◯ Animas
◯ Accu-Check
◯ Asante
◯ Medtronic
◯ Sooil
◯ Tandem t:slim
◯ Omnipod
◯ Other
◯ Prefer not to answer

If other, pump type please list

____

How much did you pay out of pocket in [currency] for your [pump_type] pump?

____
(Number only)

How much do you pay out of pocket in [currency] for a 1 month supply of insulin pump supplies?

____
(Number only)

What brand of blood testing strips do you primarily use?

◯ None
◯ OneTouch
◯ Accu-Check
◯ Abbott Freestyle
◯ Ascensia (Bayer)
◯ Precision
◯ True track
◯ True test
◯ Sanofi BG star
◯ Subscription based services (e.g. One Drop or Good Glucose)
◯ Other ______
◯ Prefer not to answer

Approximately how many test strips do you use per month?

____
(Number only)

How much do you pay out of pocket in [currency] for a 1 month supply of blood glucose testing strips?

____
(Number only)

Do you use a continuous glucose monitor/Flash Glucose Monitoring devices (CGM)?

◯ No
◯ Yes, Dexcom
◯ Yes, Medtronic
◯ Yes, Freestyle Libre
◯ Yes, other ______
◯ Prefer not to answer

How much do you pay out of pocket in [currency] for a 1 month supply of CGM/Flash Glucose Monitoring supplies?

____
(Number only)

How often do you NOT test your blood sugar due to lack of strips or CGM supplies?

◯ Never
◯ At least once per year
◯ At least once per month
◯ At least once per week
◯ Every day
◯ Prefer not to answer

Do you use or keep a glucagon emergency injection or nasal spray (Baqsimi) for hypoglycemia (low blood sugar)?

◯ Yes, Glucagon
◯ Yes, nasal spray (Baqsimi)
◯ No
◯ Prefer not to answer

You answered that you do not use or keep a glucagon or nasal spray with you. Why not?
(Choose all that apply)

☐ It is too expensive
☐ It is not available where I live
☐ I did not know it exists
☐ I do not know how to use it
☐ I do not want to keep it with me
☐ I do not feel that I need it
☐ Other ______
☐ Prefer not to answer

How much do you pay out of pocket in [currency] for a glucagon emergency shot or nasal spray?

____
(Number only)

Do you use or keep ketone strips with you?

◯ No
◯ Yes, urine strips
◯ Yes, blood strips
◯ Prefer not to answer

You answered that you do not use or keep ketone strips with you. Why not?
(Choose all that apply)

☐ It is too expensive
☐ It is not available where I live
☐ I did not know it exists
☐ I do not know how to use it
☐ I do not want to keep it with me
☐ I do not feel that I need it
☐ Other ______
☐ Prefer not to answer

How much do you pay out of pocket in [currency] for one container of ketone test strips?

____
(Number only)

The number below is the total sum in [currency] of all monthly out-of-pocket costs you've entered related to your diabetes supplies.

If it seems higher or lower than you expected, please recheck the values you have entered above.

____

How much do you pay in [currency] annually for medical/doctor visits related to diabetes? This may be in the form of a "copay," if you have health coverage.

____
(Number only)

How much do you pay in [currency] per month for your health coverage? You might know this as a 'premium.'

____
(Number only)

If comfortable, please share your total average monthly household expenses other than diabetes costs (i.e. rent/mortgage, utilities, food, leisure activities, clothing, etc).

____
(Number only)

Is there any other information you would like us to know about your diabetes care?

If you could tell your government or people in power one thing, what would you tell them?

If you would like to share more information about life with diabetes in your country, email us at contact@t1international.com.
