# Supplementary material for: Out-of-pocket expenses and rationing of insulin and diabetes supplies: findings from the 2022 T1International cross-sectional web-based survey
Source: Front Clin Diabetes Healthc. 2024 Apr 22;5:1293882. doi: 10.3389/fcdhc.2024.1293882 (PMC11070566; doi:10.3389/fcdhc.2024.1293882)
Supplement: Supplementary file 2 [file DataSheet_2.pdf]

Supplementary Material

Out-of-Pocket Expenses and Rationing of Insulin and Diabetes Supplies: Findings from the 2022 T1International cross-sectional web-based survey

Katherine Janine Souris[1*], Elizabeth Pfiester[2], Axel Thieffry[3], Yanbing Chen[4], Katarina Braune[5], Mridula Kapil Bhargava[6], Ravjot Samra[7], Pilar Gómez[8], Shane O'Donnell[9]

***Correspondence:** Katherine Janine Souris: globaladvocacy@t1international.com

1 Supplementary Tables and Figure

1.1 Supplementary Tables

Supplementary Table 1. Monthly OoPEs by healthcare coverage level

**Coverage**	**N**	**NA's (%)**	**Mean**	**Median**	**SD**
None	100	0 (0%)	345.9	201.8	455.7
Partial	501	7 (1.4%)	434.9	250.3	642.6
Full	128	7 (5.5%)	21.2	0.0	80.5

Supplementary Table 1. Descriptive statistics of monthly Out-of-Pocket Expenses (OoPEs) in USD by healthcare coverage level (Coverage), including number of participants (N); number and percent of participants not responding (NA's (%)), and mean and median (Mean and Median) and standard deviation (SD) of expenses. Supports manuscript Figure 2B.

Supplementary Table 2. Costs by category

**Category**	**N**	**NA's (%)**	**Mean**	**Median**	**SD**
Devices	731	94 (12.9)	220.1	100.0	366.1
Insulin	731	31 (4.2)	112.1	35.0	297.5
Pen needles/syringes	731	425 (58.1)	59.2	25.0	144.2
Testing supplies	731	122 (25.1)	25.1	12.2	36.0
Glucagon kit	731	318 (43.5)	57.6	25.0	93.8

Supplementary Table 2. Descriptive statistics of expense categories in USD, organized as in Supplementary Table 1. Glucose testing supplies are represented by "Testing supplies" in table. Supports manuscript Figure 2A.

Supplementary Table 3. Monthly OoPEs by country

Country	N	NA's	Mean	Median	SD
United States	453	8	471.1	269.0	689.1
Panama	34	0	434.3	340.0	292.7
Canada	41	1	252.2	192.1	282.7
India	72	0	208.0	175.5	179.3
Germany	27	0	18.2	5.5	28.2
United Kingdom	54	0	36.0	0.0	78.3
Sweden	50	1	6.4	0.0	30.9

Supplementary Table 3. Descriptive statistics of expenses in each country in USD. Organized as in Supplementary Table 1. Supports manuscript Figure 2C.

Supplementary Table 4A. Insulin rationing in the seven most represented countries

Country	Rationing Frequency	N	Total	%
United States	yearly	48	437	10.98
	monthly	30	437	6.86
	weekly or more	22	437	5.03
Panama	yearly	3	33	9.09
	monthly	3	33	9.09
India	yearly	1	61	1.64
	monthly	4	61	6.56
	weekly or more	4	61	6.56
Canada	yearly	6	41	14.63

Supplementary Table 4A. Details of insulin rationing frequencies in the seven most represented countries, including number of participants (N), number of total participants (Total) and percent of total participants within a given country reporting insulin rationing according to frequency (%). Countries with no rationing are not displayed. Supports Figure 3B.

Supplementary Table 4B. Rationing of glucose testing supplies in the seven most represented countries

Country	Rationing Frequency	N	Total	%
India	yearly	5	64	7.81
	monthly	6	64	9.38
	weekly or more	20	64	31.25
United States	yearly	64	437	14.65
	monthly	57	437	13.04
	weekly or more	41	437	9.38
Canada	yearly	10	39	25.64
	monthly	2	39	5.13
	weekly or more	2	39	5.12
Panama	yearly	5	32	15.62
	monthly	2	32	6.25
	weekly or more	3	32	9.37
Germany	yearly	2	27	7.41
	monthly	1	27	3.7
United Kingdom	yearly	2	54	3.7
	monthly	1	54	1.85
	weekly	1	54	1.85
Sweden	yearly	2	49	4.08
	weekly	1	49	2.04

Supplementary Table 4B. Detail of rationing of testing supplies in the seven most represented countries, organized as in Table 4A. Supports Figure 3B.

1.2 Supplementary Figure

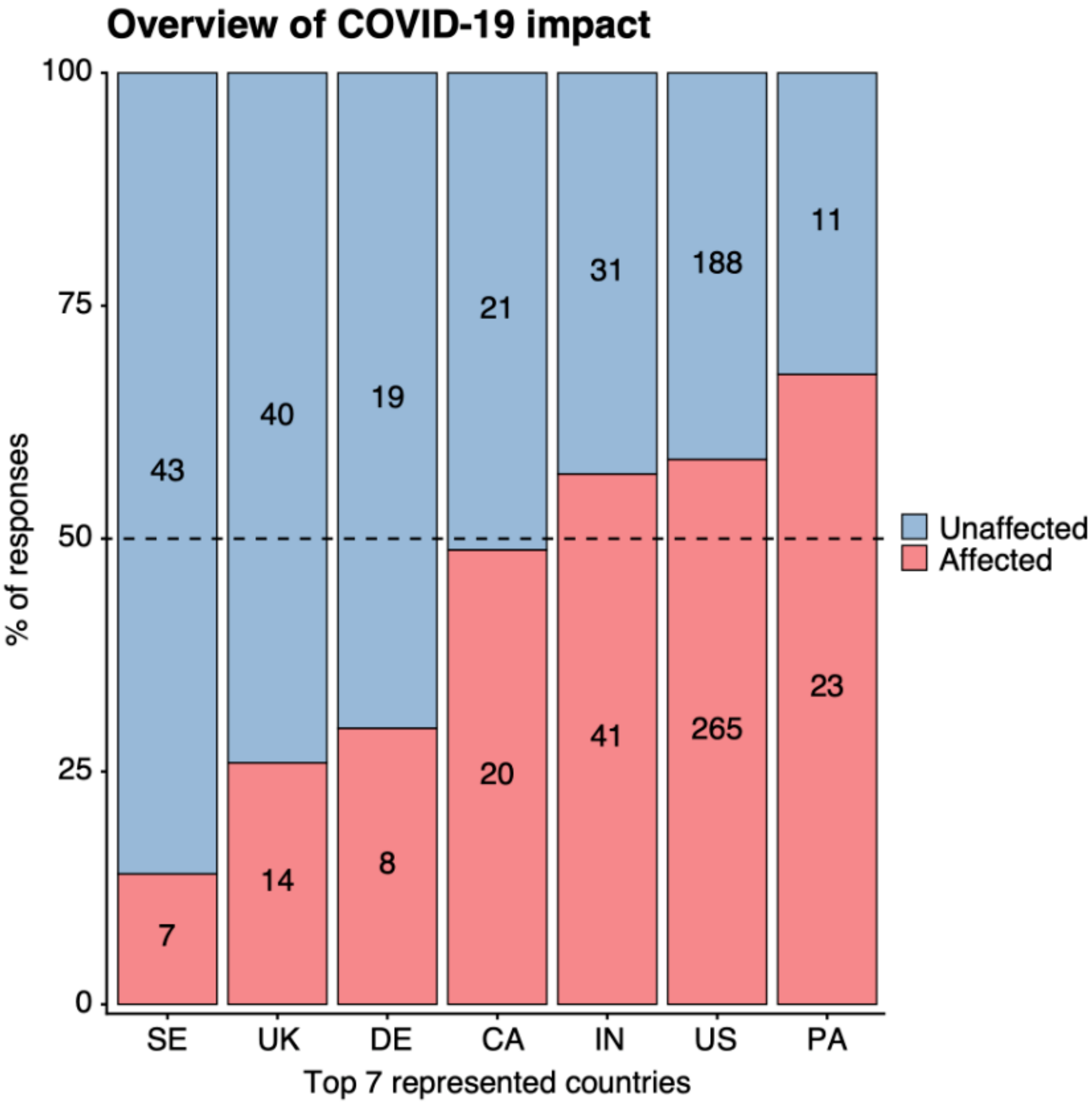

Supplementary Figure 1. Impact of the COVID-19 pandemic in the seven most represented countries

Supplementary Figure 1. Overview of respondents (Y-axis, percent) in the seven most represented countries (X-axis) reporting an impact of the COVID-19 pandemic. Number of responses are indicated within each bar.
